# The Benefits of Using a Small Caliber Ureteroscope in Evaluation and Management of Urethral Stricture

**DOI:** 10.1155/2018/9137892

**Published:** 2018-11-21

**Authors:** Walid Shahrour, Pankaj Joshi, Craig B. Hunter, Vikram S. Batra, Hazem Elmansy, Sandesh Surana, Sanjay Kulkarni

**Affiliations:** ^1^Thunder Bay Regional Health Sciences Center, Northern Ontario School of Medicine, Division of Clinical Sciences, Thunder Bay, Canada; ^2^Kulkarni Reconstructive Urology Center, Pune, India

## Abstract

**Introduction and Objective:**

The proper evaluation of urethral strictures is an essential part of the surgical planning in urethral reconstruction. The proper evaluation of the stricture can be challenging in certain situations, especially when the meatus is involved. We propose that the use of a small caliber ureteroscope (4.5 Fr and 6.5 Fr) can offer additional help and use for the surgical planning in urethroplasty.

**Methods:**

We prospectively collected data on 76 patients who underwent urethroplasties in Kulkarni Reconstructive Urology Center, Pune, India and Thunder Bay Regional Health Sciences Center, Thunder Bay, Canada. Patients had retrograde and micturition urethrograms performed preoperatively. The stricture was assessed visually using a 6.5 Fr ureteroscope. If the stricture was smaller than 6.5 Fr, we attempted using the 4.5 Fr ureteroscope. In nonobliterated strictures, we attempted bypassing the stricture making sure not to dilate the stricture. A glide wire would be passed to the bladder under vision. Stricture length, tissue quality, presence of other proximal strictures, false passages, and bladder tumors or stones would be assessed visually. If the penile stricture was near obliterative (smaller than 4.5 Fr caliber), a two-staged procedure is elected to be performed. For proximal bulbar strictures, if the urethral caliber is less than 4.5 Fr and the stricture length is less than 1 cm, we perform a nontransecting anastomotic urethroplasty (NTAU). If the stricture length is >1 cm, we perform a double-face augmented urethroplasty (DFAU). If the urethral caliber is >4.5 Fr and particularly those who are sexually active, ventral inlay with buccal mucosal grafts (BMGs) is performed. In mid to distal bulbar strictures, if the urethral caliber is >4.5 Fr, our procedure of choice is dorsal onlay with BMG. For those with urethral caliber <4.5 Fr and a stricture less than 1 cm, we perform a NTAU. For strictures longer than 1 cm, we perform a DFAU. With the exception of trauma, we very rarely transect the urethra. For panurethral strictures, we almost exclusively perform Kulkarni one-sided dissection.

**Results:**

Urethroscopy was performed in 76 patients who presented for urethroplasty from July 2014 to September 2014 (in Pune) and between April 2016 and September 2017 (in Thunder Bay). Bypassing the stricture was achieved in 68 patients (89%) while it was unsuccessful in 8 patients (11%). In all unsuccessful urethroscopies, the stricture was near obliterative <4.5 Fr. Our surgical planning changed in (13) 17% of the cases. Out of 43 bulbar strictures, the decision was changed in (9) 21% where we performed 4 DFAU, 3 AAU (augmented anastomotic urethroplasty), and 2 EAU (end anastomotic urethroplasty). In 13 penile strictures, we opted for staged urethroplasty including 3 Johansons and 1 first-stage Asopa in 30.7%. In 20 panurethral urethroplasties, 1 patient (5%) had a urethral stone found in a proximal portion of the bulbar urethra distal to a stricture ring that was removed using an endoscopic grasper.

**Conclusion:**

The use of the small caliber ureteroscope can help in evaluation of the stricture caliber, length, and tissue quality. The scope can also aid in placing a guide wire, evaluating the posterior urethra, and screening for urethral or bladder stones. It can also improve the preoperative patient counselling and avoid unwanted surprises.

## 1. Introduction

Urethroplasty with its different techniques has evolved significantly over the last three decades. Many reconstructive urologists perform formal urethrography (retrograde and antegrade) as a part of their surgical planning either preoperatively or perioperatively. Apart from the usual uses of urethroscopy in dilation, direct visual internal urethrotomy, and primary endoscopic realignment in pelvic fracture urethral injury, the use of urethroscopy is important for planning and performance of urethroplasty [1–4].

Historically, the endoscopes used have been rigid scopes of sizes ranging from 17 Fr to 22 Fr. Later, flexible cystoscopes were introduced with sizes ranging between 15 and 17 Fr. The average ureteroscope has a size of 7.5–9 Fr. The development of smaller caliber scopes (6.5 Fr and 4.5 Fr) can be of benefit to the reconstructive surgeon in their assessment of the urethral stricture without dilation or disruption of the stricture. We propose that the use of small caliber ureteroscopes (6 Fr and 4.5 Fr) can offer additional help and use for the surgical planning and technique in urethroplasty.

## 2. Materials and Methods

We prospectively collected data on 76 patients who underwent urethroplasties in two centers: Kulkarni Reconstructive Urology Center, Pune, India, and Thunder Bay Regional Health Sciences Center, Thunder Bay, Canada. These patients were undergoing various types of urethroplasties for anterior urethral strictures. We excluded patients with posterior urethral stricture. All patients had urethroscopy other than those who were excluded. Patients were consented appropriately and had retrograde urethrogram (RGU) performed preoperatively. Some of the patients had micturition cystourethrogram (MCU) done as well depending on the RGU results, feasibility, and the pathology of the disease. If the RGU was not conclusive, the stricture was obliterated, or the urethra proximal to the stricture was not properly visualized, MCU would be performed. All patients received appropriate preoperative antibiotics.

6.5 Fr ureteroscope is introduced into the meatus. The scope was passed slowly through the urethra till the stricture site. The stricture caliber and length were evaluated under vision. Assessment of the stricture was done visually to differentiate obliterative (no lumen) from near-obliterative (<4.5 Fr) and nonobliterative (>4.5 Fr) stricture. If the 6.5 Fr scope can easily bypass the stricture without dilating it, the 4.5 Fr ureteroscope was not used. If the stricture was smaller than 6.5 Fr, the 4.5 Fr ureteroscope would be used instead. After assessment of the stricture, a glide wire (0.365 mm) would be passed to the bladder under vision. Length, tissue quality, presence of other proximal stricture, false passages, and bladder for tumors or stones would be assessed visually. Occasionally, the wire passes first to guide the passage of the small caliber endoscope. In cases of near obliteration less than 4.5 Fr, the wire would be passed under vision without bypassing the stricture. If the stricture was completely obliterated and the patient had suprapubic catheter, an antegrade cystoscopy was done to evaluate the bladder, bladder neck, and posterior urethra till the verumontanum.

### 2.1. Management

**Figure 1:**
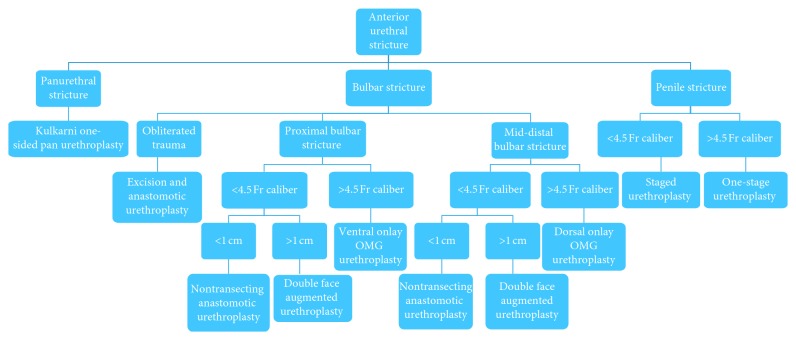
Anterior urethral stricture management algorithm (Postpost urethroscopy). BMG: Buccal Mucosal Graft.

We usually perform one-stage procedure for all different strictures. Our algorithm is presented in Figure 1. If the urethroscopy findings were different than our initial RGU, we changed our initial operative plan. If the penile stricture was near obliterative (smaller than 4.5 Fr caliber), a two-staged procedure is elected to be performed. For proximal bulbar strictures, if the urethral caliber is less than 4.5 Fr and the stricture length is less than 1 cm, we perform a nontransecting anastomotic urethroplasty (NTAU). If the stricture length is >1 cm, we perform a double-face augmented urethroplasty (DFAU). If the urethral caliber is >4.5 Fr and particularly those who are sexually active, ventral inlay with buccal mucosal grafts (BMGs) is performed. In mid to distal bulbar strictures, if the urethral caliber is >4.5 Fr, our procedure of choice is dorsal onlay with BMG. For those with urethral caliber <4.5 Fr and a stricture less than 1 cm, we perform a NTAU. For strictures longer than 1 cm, we perform a DFAU. With the exception of trauma, we very rarely transect the urethra. For panurethral strictures, we almost exclusively perform Kulkarni one-sided dissection with BMG.

## 3. Results

Urethroscopy using a small caliber endoscope was performed in 76 patients who were presenting with obstructive urinary symptoms from July 2014 to September 2014 in Pune, India, and between April 2016 and September 2017 in Thunder Bay, Canada. Patient data are presented in Table 1. Mean age was 49 years (range 22–78 years). The assumed etiologies of the strictures were instrumentation 17.1% (13 patients), lichen sclerosis 19.7% (15 patients), catheter-induced 10.5% (8 patients), failed hypospadias 11.8% (9 patients), infectious 2.6% (2 patients), and idiopathic 38.1% (29 patients). Mean stricture length was 6.3 cm (range 2–16). Stricture sites were found to be bulbar urethra in 57% (43 patients), panurethral in 26% (20 patients), and penile in 17% (13 patients). 71% (54 patients) had a previous visual internal urethrotomy with a mean of 1.6 (range 0–5). 26.3% (20 patients) had a previous urethroplasty. 35.5% (27 patients) were on self-dilation and CIC prior to referral. Successful urethroscopy was achieved in 91% (69 patients), while it was unsuccessful in 9% (7 patients). In all the 7 patients who had failed urethroscopy, they had near-obliterative strictures that were smaller than 4.5 Fr. 16 patients (21%) had strictures <6.5 Fr but >4.5 Fr.

Among those with penile strictures, 30.7% (4 patients) the operative procedure was changed from single-stage approach to 2-staged approach. Of those, 3 patients underwent first-stage Johansons and 1 patient had first-stage Asopa.

In bulbar strictures, the decision to go from ventral inlay or dorsal onlay to DFAU, NTAU, and end anastomotic urethroplasty (EAU) was observed in 21% (9 patients; 4 DFAU, 3 NTAU, and 2 EAU). In one patient (5%) with panurethral stricture, a urethral stone was encountered. The stone was found in a proximal portion of the bulbar urethra distal to one of the strictured rings. After the urethra was opened during the urethroplasty, a scope was passed and the stone was removed with a grasper. Out of the 13 patients in whom the decision was changed after urethroscopy, 8 (62%) had a near-obliterative stricture that was less than 4.5 Fr. The other 5 (38%) patients had strictures that were slightly larger than 4.5 Fr.

## 4. Discussion

The choice of the surgical technique of urethroplasty can vary among different reconstructive surgeons. While the evaluation techniques are almost universal, the complete evaluation of the clinical situation can be of crucial importance. The use of retrograde urethrogram has been the cornerstone of the evaluation of stricture disease [2, 5]. The value of the RGU is limited by the experience of the urologist and the radiologists' ability to interpret the results. The RGU usually underestimates the stricture length [5, 6]. Some reports favor sonourethrography for determining the stricture length and extent of spongiofibrosis which can help in operative planning [7–9]. Most reconstructive surgeons would concur with the use of urethroscopy for evaluation of the urethral stricture [1–4, 10]; there is no consensus on size of the scope. The use of a smaller caliber 4.5 Fr ureteroscope can provide several benefits. These benefits include the following:

1. The proper assessment of the length of the stricture even beyond the distal narrowed ring/strictured areas: the small caliber can navigate easily through a 5–6 Fr narrowed urethra without affecting the stricture or causing an unwanted dilation effect of the larger scopes. Hence, the number of proximal strictures that can be properly assessed would be increased given the smaller caliber endoscopes.
2. The proper assessment of the posterior urethra, bladder, and bladder neck contracture: as demonstrated by Hosseini et al. [1], the assessment of the posterior urethra and the bladder is very important. Identification of bladder tumour can greatly alter the disposition of the patient. In addition to that, the possible presence of urethral tumors, false passages, or diverticulae proximal to the stricture can be identified visually. These can easily be missed on RGU for either technical or clinical difficulties while performing the study. In our study, we had a patient who had a stone in the proximal portion of his long stricture. If the usual male sound was passed blindly during the procedure to assess proximal patency, this stone might have been pushed into the bladder or into the urethral mucosa creating another stricture in the future.
3. Passing the ureteroscope beyond the narrowed/strictured areas to pass a guide wire the bladder: when there is a difficulty in passing the guide wire into the bladder, the small caliber scope can help in passing the guide wire under vision. This avoids false passage placement of the wire and increased confidence of proper placement into the bladder.
4. Reinsertion of displaced Foley catheter in the early postoperative period: the small caliber scope can aid in the passage of a new catheter over a guide wire. The reinsertion of the catheter can be tricky with the aim not to cause any false passage, disruption of sutures, or damage to the graft by blind insertion of catheter or guide wire.
5. In cases of patients with combined urethral stricture and benign prostatic hyperplasia: the use of small caliber ureteroscope can help in assessing the prostate and deciding if there is a possible need for prostate surgery prior to urethroplasty.
6. Scoping the bladder and the posterior urethra is important in cases with complete urethral obliteration, or pelvic fracture urethral injury patients to rule out stones, or tumors, and to assess bladder neck and posterior urethra. While the usual size of the suprapubic catheter is around 14 Fr, the use of a small caliber scope would avoid the need to dilate the suprapubic canal since the flexible cystoscope is around 15-16 Fr.
7. Resection of postanastomotic polyp: this polyp is an outgrowth of mucosa at the site of anastomosis that could cause future obstruction of the urethra. These polyps can be assessed and resected with a small ureteroscope and laser.
8. Flexible cystoscopy is not yet available in many developing countries where stricture incidence is higher.
9. The availability of a small caliber ureteroscope could benefit in the pediatric population as well as the adult population with stricture disease.
10. The evaluation of the posterior urethra that cannot be properly assessed using the RGU and needs MCU to be assessed: MCU can be painful and difficult to perform on the patient in addition to time consumption in filling the bladder in a retrograde fashion and with increased radiation exposure. Despite repeated attempts at proper MCU, the resulting images are sometimes inadequate.

**Figure 2:**
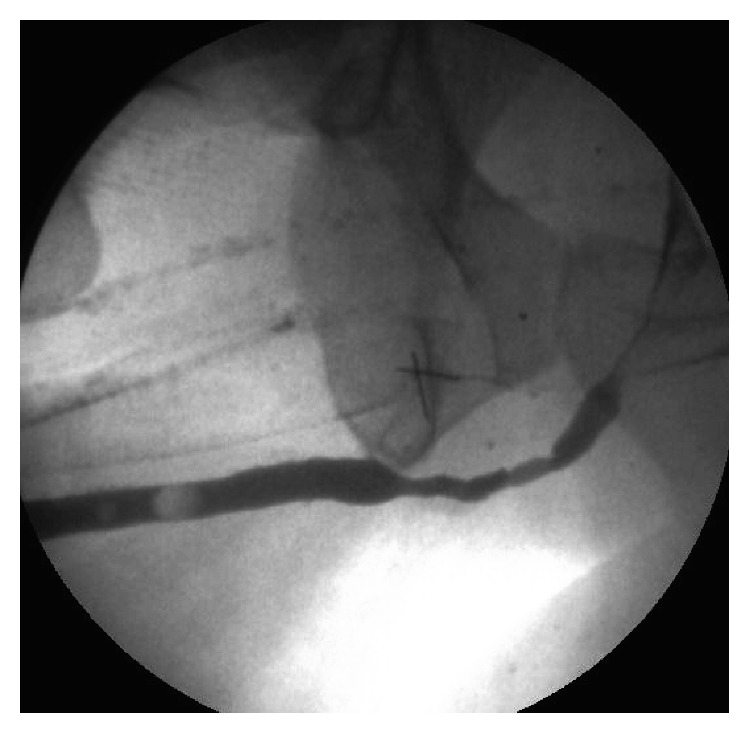
RGU demonstration of a bulbar stricture (>4.5 Fr and <6.5 Fr).

In the cases where the urethroscopy was successful, there were 16 patients (21%) who had nonobliterative (Figure 2) stricture (>4.5 Fr and <6.5 Fr) that were yet successfully evaluated because of the small caliber of the scope used. While definition of near-obliterative stricture can be loose, we aim to define it as a tight stricture that is less than 4.5 Fr in which we can see a lumen. Having the ability to properly assess the stricture and pass the guide wire safely into the bladder under vision helped in the evaluation and the surgical planning.

In cases of panurethral stricture, the urethroscopy was successful and aided in determining the length of grafts needed. The added information increased the efficiency of the operative time by providing data for the second team harvesting BMG although this has not been addressed in this study.

**Figure 3:**
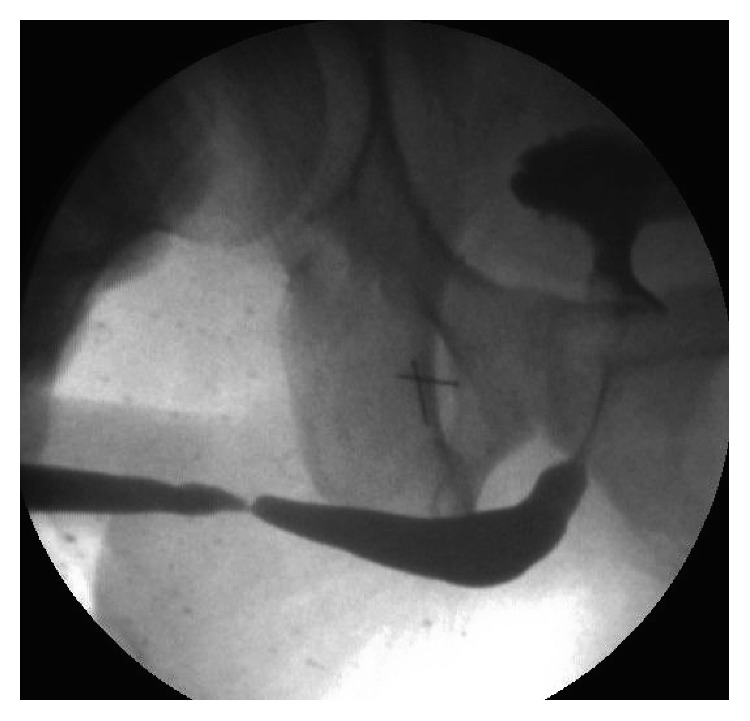
RGU of a peno-bulbar stricture that is nearly obliterative <4.5 Fr.

**Figure 4:**
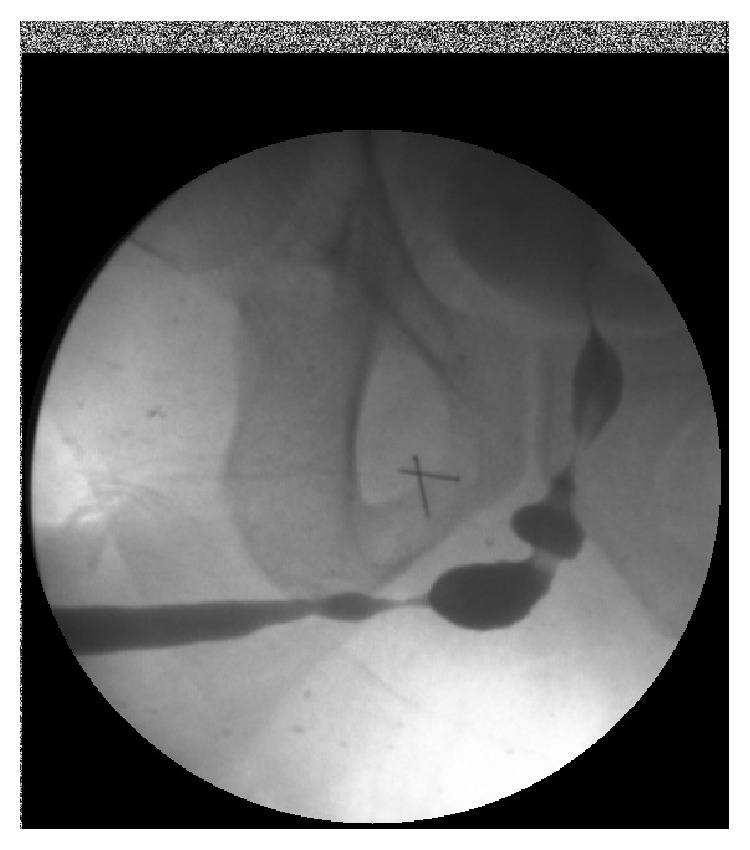
RGU of a peno-bulbar stricture that is nearly obliterative <4.5 Fr.

Urethroscopy was not successful in 8 patients (11%). The strictures were near obliterative in all of them (Figures 3-4), becoming almost like pin point strictures. In those cases, the guide wire was passed through the pin point stricture under vision but the urethra proximal to that stricture was not assessed. After the urethra was opened, the scope was passed proximally into the bladder. The change in the planned procedure was decided after the assessment of the stricture type, length, caliber, and quality of the urethra proximally and distally. Initially, the procedure was planned based on the findings on the RGU and MCU. We observed a change in the planned procedure in 13 patients (17%) after the urethroscopy. We consider this to be significant yet the impact can vary depending on the surgical technique and the algorithm used for surgical planning. We did not perform a statistical analysis as this was not the aim of our study. This would be addressed in the future with another prospective study.

RGU has limitations. An RGU for nearly obliterated urethral stricture will underestimate the caliber of the urethra proximal to the nearly obliterated portion. A proximal narrow urethra on RGU can either be secondary to a tight distal ring or a long strip of narrow urethra. Using the small caliber endoscope would allow the surgeon to go through the stricture and formulate the surgical plan. It is also not always possible to perform a voiding urethrogram in those very tight strictures unless they have a suprapubic catheter.

26% of the cases had a urethra that is smaller than 6.5 Fr that required the use of a 4.5 Fr scope. Putting the glide wire under vision in the 8 patients (11%) who had a stricture less than 4.5 Fr was important. Blind guide wire insertion might lead to false passages and worsening spongiofibrosis.

Patient population of urethral strictures might suffer from urine stasis, chronic catheterization, and retention. Hence, these patients might be at increased risk of bladder stones and tumors.

The possible criticism of urethroscopy in general is that it might cause dilation. This would be negated with the use of a very small caliber as the one we used in this study. Other possible criticisms would be the increased operative time from that extrastep. In our opinion, these extrafew minutes spent for the proper evaluation would be compensated with the proper evaluation and decision-making. The time can also be saved by deciding to harvest the graft early on or going for EAU as compared to waiting till the urethra is formally opened and the stricture is evaluated. The cost factor should always be considered. Given that the scope is always an added value to the armamentarium of reconstructive surgeons, this should not be a limiting factor.

Depending on the urology clinic setup, urethroscopy could be performed preoperatively. This would help in counselling and involving the patient in the decision-making process.

## 5. Conclusion

The use of a small caliber ureteroscope in the operative setting of urethroplasty can add value to the initial evaluation. It can also help in properly evaluating the stricture, planning the surgery appropriately, and avoiding unwanted surprises during the surgery without damaging the urethra.

## Figures and Tables

**Table 1:** Patient demographics.

Mean age	49 (22–78)
Etiology	
Instrumentation	17% (13)
1. catheter	10.5% (8)
2. lichen scleroses	20% (15)
3. Failed hypospadias	12% (9)
4. infectious	2.5% (2)
5. idiopathic	38% (29)
6. mean stricture length	6.3 cm (1–16)
Stricture location	
1. bulbar	57% (43)
2. penile	17% (13)
3. pan-urethral	26% (20)
Previous direct visual urethrotomy (DVIU)	71% (54)
Mean DVIU	1.6 (0–5)
Previous urethroplasty	26.3% (20)
Patients on self-dilation	35.5% (27)
Decision changed after urethroscopy	17% (13)
Successful urethroplasty	89% (68)
Percentage of near obliterative stricture <4.5 Fr	11% (8)
Percentage of stricture >4.5 Fr and <6.5 Fr	26% (20)

## Data Availability

The data are available between Kulkarni Center for Reconstructive Urology, Pune, India, with Dr. Sanjay Kulkarni. The data for Thunder Bay Regional Health Sciences Center are available with Dr. Walid Shahrour. Dr. Walid Shahrour has collected both sets of data, and a copy of the data is available with Dr. Walid Shahrour.
